# Factors influencing the elderly’s adoption of mHealth: an empirical study using extended UTAUT2 model

**DOI:** 10.1186/s12911-022-01917-3

**Published:** 2022-07-24

**Authors:** Jahir Uddin Palas, Golam Sorwar, Md Rakibul Hoque, Achchuthan Sivabalan

**Affiliations:** 1 School of Science and Engineering, Southern Cross University, Gold Coast, Australia; 2 Faculty of Management Studies & Commerce, University of Jaffna, Jaffna, Sri Lanka; 3 Department of Banking and Insurance, University of Dhaka, Dhaka, Bangladesh; 4 Department of Management Information Systems, University of Dhaka, Dhaka, Bangladesh

**Keywords:** mHealth, Elderly, UTAUT2, Quality of life, Service quality, Developing country

## Abstract

**Background:**

Despite the high usage of mobile phones in daily life in developing countries like Bangladesh, the adoption and usage of mHealth services have been significantly low among the elderly population. When searching previous studies, the researchers have found that no studies have empirically investigated whether the quality of life and service quality are significant for mHealth adoption by elderlies in Bangladesh. Hence**,** this study aimed to extend the Unified Theory of Acceptance and Use of Technology by adding service quality and the quality of life to empirically find the key factors that influence elderlies’ intention to adopt and use mHealth services in Bangladesh.

**Methods:**

A face-to-face structured questionnaire survey method was used to collect data from 493 elderlies (aged 60 years and above) in Bangladesh. The data were analyzed with the Structural Equations Modelling (SEM) and Fuzzy Set Qualitative Comparative Analysis (fsQCA).

**Results:**

SEM results suggested that Social Influence, Hedonic Motivation, Price Value, Habit, and Service Quality had significant impact (*p* < 0.05) on the elderlies’ behavioral intention to adopt mHealth services. Service Quality, Quality of Life, and elderlies’ Habit were found significant in explaining the Use Behavior of mHealth services. Quality of Life did not show significant (*p* > 0.05) effect on Behavioral Intention, which is inconsistent with existing literature. In addition, fsQCA findings suggest how the intensity of the influencers may contribute to high versus low m-health behavioral outcomes.

**Conclusions:**

The findings have significant implications for theory, practice and future research as explained in the paper. The originality of this study is the integration of quality of life and service quality into UTUAT2 to explain the users’ behavioural intention and use behaviour. Overall, the findings may contribute to shaping appropriate policies for designing and implementing mHealth services effectively for elderlies in developing countries.

## Introduction

With the advancement of medical science, the population of aging people is increasing more than before worldwide, especially in developing countries [1, 2]. According to WHO [3], about 10 percent of the world population is aged 60 years or more, accounting for 840 million people, and it is predicted to be more than one billion or about 12 percent by 2030 [4, 5]. It is also anticipated that five developing countries: Bangladesh, India, Pakistan, China, and Indonesia, would cover around 50 percent of the world’s elderly population by 2025 [6]. Due to a poor socio-economic condition in the developing nations of Asia, the elderly reaching their sixties or beyond struggle with inadequate diet, no or limited access to quality healthcare, and poverty [7]. On the other hand, traditional sources of security and healthcare for an aging population have already started to disappear with the changing concept of family and responsibility towards aged ones due to globalization [8].

Elderlies are comparatively more prone to physical disability, mental incapability, and chronic diseases than others [9]. They need frequent visits to the doctor and/or hospital, which is inevitable when they become older [10]. Being more vulnerable than younger people, they need to rely on social connections and family members for monitoring and ensuring compliance with medication and a healthy lifestyle [11]. However, due to their physical inability, frequent movements are difficult for them [12]. Also, due to a higher hospitalization cost, such monitoring comes out as best if it could be arranged at home [13]. Family members and relatives need to make room in their busy schedules to take care of elderlies’ health in manifold ways, e.g., doctors’ appointments, routine medication, exercise, specialized food, and so on [14].

Because of the significant financial burden, very few older couples or single elderlies can avail themselves of external caregivers; hence long-term care for them is most challenging [15]. They often need help with comprehending and administering drugs, interpreting diagnostic test reports, finding emergency contacts of hospitals, etc. Besides, in cases of chronic diseases or emergencies, they require intense monitoring round the clock. Moreover, the recent worldwide outbreak of COVID-19 manifests how older people might become the most vulnerable and subject to quarantine and follow-up care from home [16]. In such a scenario, Mobile Health (mHealth) services play a role in minimizing costs, time, stress, and discomfort [17] by overcoming the stated inconveniencies and enabling healthcare access from home [18]. Other than complex tests and operative measures, routine consultation and medication from doctors can easily be availed through mHealth [19, 20].

As mHealth can facilitate the registration for doctors’ appointments, receiving medical prescriptions, test results and treatments after diagnostic [21], it is found as a significant and efficient way of access to healthcare by elderlies [22, 23] as well as improving health condition of elderlies [24]. Recent studies have reported multiple successful applications of mHealth for elderlies, such as self-management of chronic diseases [25], self-care of asthma [26], medication adherence [27], medication safety [28], and healthcare monitoring at home [29]. Other diverse uses of mHealth for elderly healthcare include empowerment and patient-centered healthcare [30], mHealth for wheelchair users [31] and fall detection [32], and supporting elderlies in outdoor risk circumstances [33]. Overall, parallel to regular face to face healthcare, mHealth helps as an additional but significantly by playing various roles [34].

Despite potential advantages with mHealth applications, only a handful of the elderly population embraces mHealth in their real-life needs, while most still depend on traditional health services [35]. Recent studies have identified motivation, perception [36], low literacy, user interface, cost, and income [37] as the significant barriers to adopting mHealth applications. Low mHealth uptake due to these barriers is commonly reported in developing countries like India, Pakistan, Sri Lanka, and Bangladesh [38–40] that have similar socio-economic status. On the other hand, technology anxiety, resistance to change, and effort in using were also found as potential barriers in mHealth adoption by the elderly in Bangladesh [41]. In contrast, Kaium, Bao, Alam, and Hoque (2020) [42] showed that performance expectancy, facilitating conditions, and social influence are significant influencing factors for the rural older people's adoption of mHealth services in Bangladesh [42]. Another study found that social influence, facilitating condition, performance, and reliability influence the general population's behavioral intention to use mHealth services in Bangladesh [43].

However, no previous studies have empirically investigated whether the quality of life (QL) and service quality (SQ) are significant factors for mHealth adoption by elderlies in Bangladesh. The researchers assume that if the quality of life (QL) and service quality (SQ) are integrated into the Unified Theory of Acceptance and Use of Technology (UTAUT2), unlike any previous studies, the extended theoretical framework can explain the adoption of mHealth by elderlies more with new findings which may have significant implications in the context of developing countries. Therefore, this study aimed to extend the Unified Theory of Acceptance and Use of Technology (UTAUT2) by adding service quality (SQ) and the quality of life (QL) to empirically find the key factors that can influence elderlies’ intention to adopt and use mHealth services. Overall, this study will answer the two research questions below.

1. What are the significant factors for mHealth adoption by elderlies in Bangladesh?
2. Does an integration of quality of life (QL) and service quality (SQ) improve the prediction power of UTAUT model in explaining the mHealth adoption by elderlies in Bangladesh?

## Literature review

### Quality of life

**U**nlike in the past, the change in lifestyle and pandemic (COVID-19) impact in Bangladesh has recently created an enormous demand for mHealth apps or websites [44]. Although not in a mature stage [45], the availability of mHealth services and their adoption are gradually increasing. Subsequently, the factors related to lifestyle (e.g., quality of life) and service (e.g., service quality) have become important for the broader adoption of these technologies.

Quality of Life (QL) is extensively studied as a significant outcome variable in the existing literature on health, information systems, and marketing. In contrast, few studies highlighted QL as a significant determinant of the intention to continue using Assistive technologies by older people [46], intention to use mHealth [47, 48], and technology acceptance for telecare program by older people [49]. A study by Shen [50] indicates that QL theory-related constructs 'Loneliness in Real Life' and 'Life Dissatisfaction' are significant antecedents of seeking support via social networks. Chen and Chan [51] indicate that constructs such as cognitive ability, social relationships, attitude to life and satisfaction, and physician functioningare related to QL and those have an impact on the acceptance of gerontechnology by elderlies in Hong Kong. Another study by Vululleh [52] found that QL is a strong determinant of behavioral intention to use eLearning technology. Although QL influences users’ acceptance of technology, few studies are available evaluating the predictive power of QL in the acceptance of mHealth technology, particularly by developing nations with low Wellness Index (WI) such as Bangladesh (WI = 43) [53].

### Service quality

Service quality (SQ) is a significant factor for healthcare, including mHealth adoption [54–57]. Inferior service quality is a major reason for discontinuing using mHealth services [58]. Existing studies confirm that service quality is multi-dimensional [59] and context-specific [60]. Most studies focus on system and information quality dimensions such as the accessibility and reliability of service delivery infrastructure, information privacy, and trust. For example, Nisha et al. [61] proposed a conceptual model to examine the factors primarily related to system quality and information quality to understand users’ intention to use mHealth services in Bangladesh. Alam et al. [43] introduced perceived reliability of service delivery infrastructure and data privacy as an additional construct with the base UTAUT constructs to identify key factors affecting the mHealth adoption by the highly educated (100% graduate or above), whereas about 65% of the older population in Bangladesh have only primary education [62] and younger (majority, 73% aged 55 years or less) users (patients) in Bangladesh. Similarly, Kaium et al. [42] investigated the reliability of the technical aspects of service provision infrastructure and found no significant impact on mHealth adoption by the rural population in Bangladesh.

Further, Jandavath & Bryan (2016) [63] argued that among other dimensions, empathy has significant effects on behavioral intention to adopt healthcare services. A recent study by Zobair et al. [64] indicates that the health staff motivation in the caring and individualized attention has substantial effects on the quality of care and patient satisfaction in telemedicine service in Bangladesh. In another qualitative study by Khatun et al. [65] found that general people (aged between 18 and 63yrs) are concerned about the quality of healthcare providers in mHealth services in Bangladesh.

While previous research suggests that quality of life (QL) is also positively associated with health behaviour but has not been studied much to understand how it influences mHealth technology acceptance, specially by older populations, who are susceptible to various chronic health issues that may worsen their QL. Until now, there are no studies available investigating the factors influencing the adoption of mHealth services by the elderly from a human-centric perspective: the influence of health care providers' e. g., general practitioner involved, motivation, medical advice (service) quality, users trust in the service providers, the competence of provider to deliver service over mobile technology and their empathy.

### Extension of UTAUT

As a theoretical framework, UTAUT has been extended and used in many studies to investigate a broader range of factors that might influence the adoption and usage of mHealth services by elderlies. For instance, Hoque & Sorwar [41] used the Unified Theory of Acceptance and Use of Technology (UTAUT) to study the factors influencing adoption and usage of mHealth from older population perspective in the Bangladesh context. They extended the UTAUT model with two additional variables, i.e., technology anxiety and resistance to change for understanding the users’ behavioral intention to adopt mHealth services. The extension of UTAUT is practiced by many previous studies related to mHealth, such as Shiferaw et al. [66] extended UTAUT by adding self-efficacy and attitude for understanding healthcare providers’ acceptance of mHealth. Similarly, Khan et al. [67] extended UTAUT by adding perceived ubiquity, perceived trust, and technology anxiety to study whether older adults can use mobile health (mHealth).

Besides, in a recent study in the context of Bangladesh as a developing county, Moudud-Ul-Huq, Swarna, and Sultana [68] used an extended version of UTUAT to find the factors that may significantly affect the elderly’s intention to use mhealth services. Likewise, Alam et al. [69] added self-quarantine and health consciousness to extend UTUAT when studying the factors important for mHealth adoption. In another previous study, Alam et al. [70] extended UTUAT to test whether trust, privacy, self-efficacy, and lifestyle in addition to the core UTUAT factors, influence the adoption of mHealth apps in Bangladesh. Alam et al. [70] also integrated important factors from IS Success Models into UTAUT2 to understand whether mHealth improves the quality of healthy life in a developing country context.

However, none of the studies above extended UTUAT by adding SQ and QL to investigate the important factors in mHealth adoption, especially targeting the elderlies in developing countries. Therefore, it is imperative to investigate the impact of SQ and QL to uncover a more generalized and widely applicable model for understanding the users' behavioral intention of mHealth by elderlies in the context of developing countries like Bangladesh.

## Theoretical framework and hypotheses

To attain the research objectives as stated in the previous section, this study adopted the most used and influential user acceptance and usage analysis model, namely, Unified Theory of Acceptance and Use of Technology (UTAUT2) [71]. To explain the intention to use and use behavior of technologies, a number of theories have been developed over time, such as the Theory of Reasoned Action [72]; the Technology Acceptance Model [73]; the Social Cognitive Theory [74]; the Model of PC Utilization [75]; the Theory of Planned Behavior [76]; the combined TAM-TPB model [77]; the Motivational Model [78] and the Innovation Diffusion Theory [79]. With a comprehensive study of the models mentioned above, the researchers have found that the UTAUT2 model has all the user acceptance factors found in the TAM, TRA, SCT and TPB models [80].

In other health related studies, including e-health, UTAUT has been widely used, and researchers claim that the model can explain up to 70% of the users' intention variance [46, 81, 82]. UTAUT2 extended the UTAUT model with three additional factors, namely, Hedonic Motivation, Price Value, and Habit, whereas the basic UTAUT model includes Performance Expectancy, Effort Expectancy, Social Influence, and Facilitating Condition [49]. Here, all seven factors are hypothesized to have an influence on the behavioral intention to use technology. However, the "Habit” factor is modelled as having influence on both behavioral intention and use behavior. Finally, the model tests the influence of behavioral intention on the use behavior of technology.

**Fig. 1:**
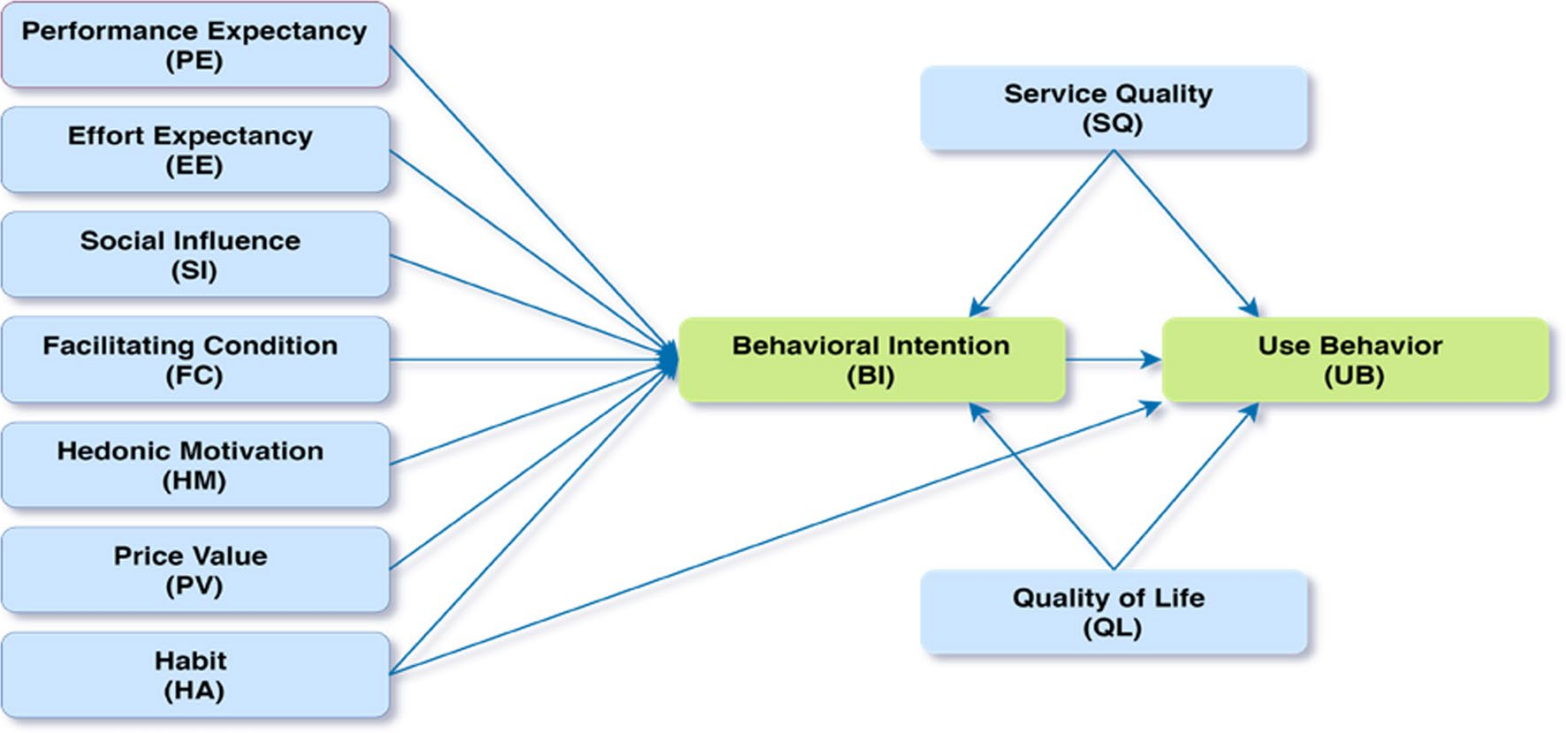
Conceptual research model. Performance expectancy = PE, Effort expectancy = EE, Social influence = SI, Facilitating condition = FC, Hedonic motivation = HM, Price value = PV, Habit = HA, Service quality = SQ, Quality of life = QL, Behavioural intention = BI, Use behavior = UB

In addition to the constructs of UTAUT2, two important determinants of user intention and use behavior, namely the service quality (SQ) and quality of life (QL) [46, 49, 83, 84], have been added in the proposed model (Fig. 1). QL was considered as a perception factor for elderlies to adopt assistive technology [85], and a significant relationship was found between perception towards QL and technology acceptance of elderlies in the telecare program [84]. In mHealth literature, SQ has been considered as an influential factor that plays a pivotal role in health systems [83, 84]. While explaining the adoption intention and use behavior, SQ has been found to have a strong positive impact [77, 86].

### Performance expectancy (PE)

As per Venkatesh et al. [87], performance expectancy relates directly to the job performance of any individual and his/her belief about the extent to which the usage of a particular system will help gain performance criteria. Performance expectancy has been found to possess the strongest influence on a person's intention to adopt a particular system [41]. In healthcare information systems, PE has influence on the users’ behavioral intention [88], and on the other hand, PE exerts a direct positive effect on the user's intention to use mobile devices [77, 89]. With greater PE, users will be more likely to adopt mobile health services [90]. Thus, the following hypothesis was developed on the basis of the related literature:

#### H1

PE has positive impact on the elderly's intention to use mHealth.

### Effort expectancy (EE)

Effort expectancy is explained as the extent of ease the users feel about the system while using it [91]. Literature supports the view that EE exerts a strong impact on the users’ intention to adopt and use health information systems. As evident from Lee and Rho [88], EE was found to have direct influence on user’s intention for using mobile health monitoring system. Clinical decision support systems [41], eHealth services [77], and mobile health services [88, 92] through smartphones are some of the important study findings that support that EE has strong influence on the user's intention to adopt mHealth services. Therefore, the hypothesis was formed as:

#### H2

EE has positive impact on the elderly's intention to use mHealth.

### Social influence (SI)

Social influence has been defined as the belief of the important ones to an individual about his/her using a new system [93]. SI has been found instrumental in users’ intention to adopt technology [94], to use health related mobile services [95], to use digital information in healthcare [41], to capture users’ overall perception of mHealth services [77], in consumers’ acceptance of online pharmacies [96] and in explaining elderlies’ adoption of mHealth services [97]. Based on the discussion, the hypothesis was derived as:

#### H3

SI has positive impact on the elderly’s intention to use mHealth.

### Facilitating condition (FC)

Facilitating condition (FC) is explained as the availability of organizational and necessary technical infrastructure to facilitate the system usage [88]. As evident from Mun et al. [94], FC was found to have direct influence on user’s intention to use technology. User’s adoption and usage of health information systems [98] and mobile health services [99] through smartphone are found to be strongly linked with FC. Facilitating conditions have been significant in understanding users’ overall perception of mHealth services [100] and elderlies’ acceptance behavior of telehealth services [101]. Therefore, the hypothesis was formed as:

#### H4

FC has positive impact on the elderly’s intention to mHealth.

### Hedonic motivation (HM)

This variable signifies the inherent fun, enjoyment, or pleasure an individual derives while using technology, and such motivation is found significant in explaining the adoption and usage of technology [101–103]. From the consumers' perspective, hedonic motivation plays a significant role in influencing adoption intention and usage of technology, as evident in the existing Information System (IS) literature [104, 105]. In chronic illness management, hedonic motivation has also been significant in the adoption of an online-based self-management support system [106]. Moreover, hedonic usage of smart home technologies was found to have great potential to retain/improve mobility, physical health, and overall well-being of inhabitants, including elderlies [107]. Thus, the hypothesis was posited as:

#### H5

HM has positive impact on the elderly’s intention to use mHealth.

### Price value (PV)

From a generic view, while usage of technology implies costs in financial terms, monetary compensation has significant impact on users' adoption and, most importantly, the continued usage. Therefore, price value can be defined as the perceived usefulness a consumer receives from using a mHealth service against the monetary cost paid for availing that particular service [108]. When a user perceives benefits that exceed the monetary costs paid, the price value is positive, which ultimately exerts a positive impact on the intention to use technology. Price value has significant impact on the users' intention to use health and fitness applications [108]. Therefore, the hypothesis concerning price value was formed as:

#### H6

PV has positive impact on the elderly’s intention to use mHealth.

### Habit (HA)

Habit is people's tendency to perform a task or behavior or use technology because of experience of usage and learning [107]. On the other hand, Kim and Ban [109] explained habit as automaticity. Habit has been identified as the prior behavior [60] and the degree to which an individual perceives that the behavior intention or usage is automatic [110]. Experiences and feedbacks of past usage exert influence on beliefs and, as a result, on the next behavioral performances. Habit, as a psychological side of BI, has been a meaningful construct to influence behavioral intention (BI) and use behavior (UB) and use behavior (UB) [111]. Therefore, the following hypotheses were developed:

#### H7

HA has positive impact on the elderly’s intention to use mHealth.

#### H8

HA has positive impact on the elderly’s use behavior of mHealth.

### Service quality (SQ)

Service quality is defined as the user’s judgment of the superiority or excellence of a service [60]. It is difficult to generalize the exact dimensions of service to measure its quality; however, most studies confirm that the measurement items should be multidimensional [84, 112] and specific to the concerned context [85]. Due to retaining the nature of service, service quality is significant to all service businesses [84] and specifically relevant in explaining the usage and adoption of mHealth services. In mobile health services, service quality is considered an influential element [84], and it performs a vital role in healthcare systems [82]. Service quality has a strong positive impact on user’s adoption intention and continuance intentions to use [113–115]. Considering the above discussion, the following hypotheses were developed with service quality:

#### H9

SQ has positive impact on the elderly’s intention to use mHealth.

#### H10

SQ has positive impact on the elderly’s use behavior of mHealth.

### Quality of life (QL)

According to [114], the extent of wellbeing and feeling of happiness for an individual is referred to as Quality of Life (QL). As the context of this study is specific to healthcare, the sense of wellbeing of an individual from an all-round perspective is captured in the definition of QL [82]. Extant literature of both information systems [116] and marketing [117] reported QL predominantly as a significant outcome variable. However, a study by Akter et al. [82] found that QL has significant impact on users’ Continuance Intentions (CI) to use mHealth service. QL has significant impact on the perception relating to a technology’s usefulness [46] and is believed to be an important technology concern for individuals of feminine cultural values [49].

While testing the cultural dimensions of masculinity and feminism, QL has been found as an important factor in behavioral intention to use technology [50]. Specific to the elderlies' perceived QL has been considered as a salient perception factor having impacts on the adoption of assistive technologies [118]. Chou et al. [49] identified significant relationship between perception towards QL and technology acceptance of elderlies in a telecare program. As per the above discussion, QL has been designated as an influencing factor for behavioral intention and mHealth usage. Such an alternative approach is critical to enhance understanding of mHealth adoption and usage because of an individual’s concurrent lifestyle. QL may have important roles to play in the mHealth service adoption and continued use, as evidenced by Shen [74] and Alaiad [73]. Thus, the hypothesized relationships were:

#### H11

QL has positive impact on the elderly’s intention to use mHealth.

#### H12

QL has positive impact on the elderly’s use behavior of mHealth.

### Behavioral intention (BI) and use behavior (UB)

Behavioral Intention has a strong positive relationship with Use Behavior, as documented in a number of studies [119]; [77, 120, 121]. In both cases of technology [77] and health information systems [86], BI is a strong predictor of UB. In addition, physicians’ BI has been significantly influenced by their personal innovativeness [87]. Based on the study findings, the following hypothesis was developed:

#### H13

BI has positive impact on the elderly’s use behavior of mHealth.

The factors and hypotheses of this study are summarized in Appendix 1.

## Methodology

The methodology in this study is based on a positivism paradigm that helps a researcher address the research problem by using a collection of data used to test hypotheses empirically. Accordingly, the deductive approach was followed based on a theoretical framework. Subsequently, while the methodology was survey research, the method was quantitative involving sampling and measuring using valid tools.

### Setting the context

This study has selected Bangladesh as a research context as it is a fast-developing county. Studies show that the rate of the older population is growing in Bangladesh faster [6, 122]. As reported by the Bangladesh Bureau of Statistics [6], elderlies aged 60 years or more accounted for nearly 6.90% of the total population. By 2025, this figure is projected to reach 9.30% [122], and by 2050, one in every five persons will be elderly in Bangladesh [7]. More than 95% of the elderly experienced health related complications [7], and the majority reported more than one health problem in Bangladesh [123].

A notable reduction in traditional support systems for elderlies is evident in recent decades in Bangladesh [7]. With the rapid urbanization and costlier living standard, families lose the financial capability to support the elderly in the old age [7]. Therefore, the old-age problems for the older generation are compounded further with the erosion of the willingness and abilities of families to support aged ones. Financial insecurity, negligence of family members, and improper health facilities are a few of the numerous problems faced by elderlies in Bangladesh [7].

While studying the macro aspects of aging in Bangladesh, Khan and Lesson [7] found that government expenditure increases in line with the growth in aging population, mostly because of support provided for health services, social facilities, and income subsidies [123]. Historically, family and society took the responsibility to manage the care necessary for the older population of Bangladesh [7]. The scenario is expected to deteriorate more in the coming days as the elderly is predicted to be about 50% of the total population by 2050 [124]. In addition, mitigating the impact of COVID-19 pandemic on the healthcare system, the broader application of mHealth (e.g., tele-pharmacy) for elderlies is expected to grow in the near future [125].

The number of mobile phone users has increased rapidly in recent years in Bangladesh. It is reported [126] that 87% of the Bangladeshi households use mobile phones. As per the Bangladesh Telecommunication Regulatory Commission (BTRC) [31, 127], the number of mobile phone users reached nearly 166 million at the end of February 2020. The facts and figures clearly indicate that Bangladesh is a potentially promising place for mHealth initiatives to improve the overall healthcare standard [31].

According to Kay et al. [128], World Health Organization (WHO) considers Bangladesh as a member of the list of 15 countries where mHealth initiatives are being used for raising health awareness. For instance, the use of mobile app “Aponjon” was recently reported positively in improving awareness and practices related to maternal and newborn health care [129]. Until now, several commendable mHealth initiatives are operating in Bangladesh, for examples, Medinova Telemedicine, TRCL (eClinic24, AMCARE, Health services for expatriates), Amader Gram, JBFH Telemedicine, mPower, Grameenphone Health Line (789), Banglalink Health Link (789), Airtel Health Portal (2,223,441), Robi Health Tips (789), TeletalkShashtho Sheba (789), JHSPH (mCARE, mTIKKA, MJiVitA), CRP Telemedicine, infoLADY (DNet), Aponjon (MAMA Bangladesh) by Dnet, and Manoshi (BRAC). In addition, a review on the Bangladeshi mHealth apps shows that privately maintained low-scale programs (e.g., Doctorola) are growing in the country [130].

In Bangladesh, most private mHealth programs are profit-oriented [131] and located in a scattered manner across the country. Each of these initiatives has its own target population with respect to age group, location, and disease specification. Most of the mHealth services mentioned above follow a generic approach to deliver their services where the service seeker needs to place a call to a certain number, e.g., 789 for "Grameenphone Health Line" (rebranded as "Tonic" recently) to consult with professional doctors about their health-related issues (Grameenphone Tonic—https://www.mytonic.com/bn/tonic-benefits). mHealth services in Bangladesh are primarily used for instant health advice, including advice on health management, offering prescriptions, suggesting referrals, and remote diagnosis by imaging and video conferencing [81, 132]. A study by Hernandez [133] indicated that lack of access and awareness are the significant barriers to use mHealth in Bangladesh. Therefore, this study is significant as it particularly aims to find the barriers and factors affecting the mHealth adoption by elderlies in Bangladesh.

### Questionnaire design

The latent constructs in the proposed model were measured with items captured predominantly from prior studies. The items were modified to fit in with the study context. The items to measure PE were adapted from [120] and [81]. Measurement items for EE, SI, and BI were captured from [81] and [84]. The questionnaire items to measure constructs, namely FC, HM, PV, and HA, were adapted from Sripalawat et al. [60] and Venkatesh et al. [81]. The constructs for SQ were adapted from Akter et al. [134], Dagger et al. [135], Parasuraman et al. [136]. The World Health Organization Quality of Life (WHOQOL)-BREF has been consulted to develop constructs for measuring the QL of respondents [137]. However, the authors developed the items for UB based on the commonly offered mHealth services by different mHealth care providers in Bangladesh (i.e., Grameenphone—Tonic, Robi- mDaktar, Banglalink—Healthlink, Teletalk—Shashtho sheba, DGHS. As per Khatun et al. [65], the four most basic services: prescription of drugs, appointments of the doctor, emergency medical services, and health counselling were selected to get maximum responses from sample respondents.

A structured questionnaire in English was preliminarily developed, and a professional translator, skilled in Bengali language, was appointed to translate the questionnaire. A pool of experts, including university academics, mHealth service providers, and medical experts, examined each question of both language versions to make sure that the questions infer similar meaning. The responses to the questions were collected by using a 5-point Likert scale, starting with (1) Strongly disagree to (5) Strongly agree.

A sample of 15 was randomly selected from Dhaka Medical College and Hospital (DMCH), Bangabandhu Sheikh Mujib Medical University (BSMMU) and BIRDEM General Hospital (Shahbagh, Dhaka) hospitals for conducting a pilot study. The feedback from the pilot study guided the design of the final questionnaire and testing its effectiveness.

### Data collection

The researchers attempted to capture a sample population that was potentially representative of the entire elderly population of Bangladesh. A face-to-face survey interaction through the convenience sampling method has been followed while selecting elderly respondents. The field investigators personally distributed the questionnaires. The interviewers were trained to conduct the interviews. To be included in this study’s sample, a participant had to be (a) of age 60 years and above, and (b) a user of any mHealth service. Because, in terms of age, this study adopted the commonly used definition of elderly from the United Nations and World Health Organization, i.e., age 60 years or beyond (http://www.who.int/healthinfo/survey/ageingdefnolder/en/).

The authors approached 24 hospitals in 8 major cities in 8 divisions in Bangladesh and sought permission to recruit potential participants from the outpatients in the hospital premises. To ensure reliability, the study questionnaire included a provision for the respondents' postcode, which allowed the authors to examine if the sample covered the major geographical regions of Bangladesh. The researchers sought explicit consent from the respondents to participate in the study. To preserve individual privacy, the name of the respondents was not recorded but only the demographic information with a code number. The percentage of response rate was 94%. Due to the face-to-face survey, the study’s response rate was high. The interviewer let the potential participants aware of their right to refrain from participating at any time during the study period without having any negative implications.

The majority (60%) of the study's sample belongs to elderlies over 65 years, making the sample comparable to the elderly population above 65 years. We have used G*Power 3.1.9.7 version software for power analysis to determine the sample size, where the significance level was 0.05 (which is normally chosen in the field of IS), and the effect size was 0.025, and power was 0.95. The software shows that the minimum required sample size is 435. Furthermore, the biases and sampling errors were minimized by increasing the sample size.

### Data analysis

This research included two distinct analytical techniques: structural equation modeling (SEM) and fuzzy set qualitative comparative analysis (fsQCA). SEM was utilized to forecast the causal association between the independent and dependent variables. The traditional analytical technique, namely SEM, is significant; yet, because it is focused on net effects, it is insufficient for comprehending complicated relationships, such as configurational effects [138]. The study of how each independent variable is related to the dependent variable is known as “net effects.” The net effects technique of analysis cannot confirm how the combinations of independent variables impact the outcome variable. FsQCA, on the other hand, is a configurational analysis that investigates the combinations of predictors that lead to an outcome variable [138].

To explore the configural effects of antecedents on the outcome variables (i.e., behavioural intention towards m-health and usage behaviour of m-health), fsQCA analysis was employed. As a result, the analysis was carried out in two essential phases utilizing fsQCA software: calibration and counterfactual analysis [139]. Calibration is the process of converting the values of conditions and outcomes into membership scores ranging from 0 to 1 [139] (Ragin 2008). Agevall (2008) [140] proposes three-value fuzzy sets: the threshold for full membership (fuzzy score = 0.95), the threshold for full non-membership (fuzzy score = 0.05), and the cross-over point (fuzzy score = 0.5). In this regard, this study used the anchor values of 5 (strongly agree), 3 (neutral) and 1 (strongly disagree) as a full membership, cross-over point and full non-membership, respectively [138, 141].

In the counterfactual analysis, truth tables were produced using calibrated fuzzy set membership scores of m-health intentions and usage behaviour, as well as their antecedents. Then the truth table was refined as per the frequency (≥ 2 for medium-to-large sample size) and consistency (≥ 80) criteria. As a result, the refining process generates a list of causal antecedent configurations (i.e., necessary and sufficient conditions) for the outcome variables: m-health intention and usage behaviour. Necessary conditions are those that must exist in order for the outcome to occur, whereas sufficient conditions are those that always result in the outcome [139].

To this end, two probability metrics were calculated: coverage and consistency. Coverage denotes how many cases with the outcome are represented by a certain causal condition. The degree to which a causal combination leads to an outcome is represented by Eq. 1 below, where X is the membership score in causal combination and Y is the membership score in the outcome set.

1 $${\text{Coverage}} = \frac{{\sum {\text{min}}\left( {X,Y} \right)}}{\sum Y}$$

About consistency, Ragin (2008) [139] suggested to establish different consistency thresholds for necessity and sufficiency analyses. Equation 2 below represents consistency. For the need condition, a consistency benchmark of at least > 0.90 is recommended, whereas for the sufficiency condition, a reasonably well-established consistency standard of at least > 0.80 is recommended.

2 $${\text{Consistency}} (X < Y) = \frac{{\sum {\text{min}}\left( {X,Y} \right)}}{\sum X}$$

## Results

### Characteristics of sample

The study distributed 530 questionnaires and received 498 completed, comprising a response rate of 94%. While screening data for analysis, 493 questionnaires were finally selected after deleting outliers (3 respondents), imputing missing values (1 respondent), and removing responses of unacceptable variance (1 respondent). Table 1 presents the respondents’ demographic and other characteristics:

**Table 1:** Demographic and socio-economic characteristics of respondents (n = 493)

Variables	Categories	Frequency	Percentage (%)
Gender	Male	417	85
	Female	76	15
Age	60–65 years	199	40
	66–70 years	235	48
	71–75 years	53	11
	Above 75 years	6	1
Education	Primary	110	22
	Secondary	59	12
	Higher secondary	80	16
	Honors/Degrees	49	10
	Master’s	134	27
	Illiterate	61	13
Current living status	Accompanied by family Members	366	74
	Couple only	103	21
	Alone	24	5

### Assessment of measurement model

According to Hair et al. [136], the measurement model needs to be assessed for internal reliability and discriminant and convergent validity to confirm the model fit for the study. In this study, Cronbach’s alpha (α) and composite reliability (CR) were used to evaluate internal reliability. A Cronbach’s alpha (α) and the composite reliability (CR) value of 0.70 for the constructs is the acceptable level of internal consistency in a model [142]. Assessing the convergent validity, average variance extracted (AVE) is used with items’ loading at least 0.50 to ensure construct validity [143]. Table 2 presents the AVE, composite reliability (CR), and Cronbach's alpha (α), and Appendix 2 reports the items’ loadings. The estimated items’ loadings were much higher than the benchmark of 0.50 [82]. The AVE values for all the constructs ranged from 0.626 to 0.906 exceeded the recommended level. Therefore, the conditions for convergent validity are adequately satisfied in the study. The range for Cronbach’s alpha (α) and the composite reliability values were 0.784 to 0.968 and 0.855 to 0.974, respectively, which provides strong internal reliability.

**Table 2:** The measurement model

Factors	Cronbach's alpha	Composite reliability	Average variance extracted (AVE)
Behavioral intention (BI)	0.948	0.967	0.906
Effort expectancy (EE)	0.941	0.957	0.849
Facilitating conditions (FC)	0.784	0.855	0.664
Habit (HA)	0.926	0.953	0.872
Hedonic motivation (HM)	0.918	0.948	0.859
Performance expectancy (PE)	0.966	0.972	0.853
Price value (PV)	0.929	0.955	0.876
Quality of life (QL)	0.914	0.930	0.626
Social influence (SI)	0.902	0.938	0.836
Service quality (SQ)	0.968	0.974	0.863
Use behavior (UB)	0.912	0.939	0.793

Performance expectancy = PE, Effort expectancy = EE, Social influence = SI, Facilitating condition = FC, Hedonic motivation = HM, Price value = PV, Habit = HA, Service quality = SQ, Quality of life = QL, Behavioural intention = BI, Use behavior = UB

The model’s discriminant validity was assessed through Heterotrait-Monotrait Ratio (HTMT). According to Kline [144], to have a satisfactory level of discriminant validity, the HTMT value should be lower than the value of 0.85. Table 3 shows the results of HTMT where all the values passed the HTMT 0.85 and the HTMT does not indicate discriminant validity problems.

**Table 3:** Heterotrait–Monotrait ratio (HTMT)

	BI	EE	FC	HA	HM	PE	PV	QL	SI	SQ	UB
BI											
EE	0.649										
FC	0.151	0.190									
HA	0.835	0.712	0.096								
HM	0.502	0.713	0.087	0.570							
PE	0.612	0.824	0.261	0.697	0.644						
PV	0.841	0.783	0.096	0.810	0.758	0.732					
QL	0.470	0.260	0.370	0.339	0.286	0.197	0.344				
SI	0.671	0.751	0.197	0.688	0.614	0.821	0.792	0.249			
SQ	0.637	0.480	0.192	0.501	0.436	0.365	0.521	0.755	0.399		
UB	0.344	0.355	0.246	0.349	0.328	0.340	0.338	0.268	0.265	0.636	

Performance expectancy = PE, Effort expectancy = EE, Social influence = SI, Facilitating condition = FC, Hedonic motivation = HM, Price value = PV, Habit = HA, Service quality = SQ, Quality of life = QL, Behavioural intention = BI, Use behavior = UB

Moreover, we have tested multicollinearity before assessing the structural relationships. Multicollinearity occurs when two or more independent variables are highly correlated with one another. Variance Inflation Factors (VIFs) score of an independent variable represents how well the variable is explained by other independent variables. If the VIFs results are less than 10, there is no multicollinearity. The VIFs (Appendix 3) results are lower than 10, which indicates that multicollinearity is not an issue in this study.

### Assessment of structural model

#### Overall fit of the estimated model

We have used Standardized Root Mean Square Residual (SRMR) to explain Model robustness [143]. SRMR measures the index of the average of standardized residuals between the observed and the hypothesized correlation matrices. A value less than 0.08 is generally considered a good fit [145]. Therefore, with the SRMR value of 0.071, the hypothesized model was found a good fit.

#### Coefficient of determination (R2)

The assessment of coefficient of determination (R2) is a significant part of structural model evaluation. The value of 0.7, 0.5 and 0.25 are often used to describe a strong, moderate and weak coefficient of determination [146]. The PLS Path model found that the nine exogenous constructs can jointly explain 84.5% of the variance of the endogenous construct behavioural intention. So, the overall R2 is found to be strong. The PLS-SEM also reveals that R2 for other constructs such as habit, service quality, quality of life and behavioral intention explain 46.7% of use behavior’s variances.

### Hypotheses testing

To identify the hypothesized relationships among the factors in the study, a structural model was developed and tested by path coefficients (β), *t*-statistics, and *p*-values. The results of partial least square modelling, run through SmartPLS, for the study’s structural model, are presented in Table 4 below. According to the table, all the proposed hypothesized relationships between dependent and independent variables were supported, except for the positive relationships between PE and BI (H1), EE and BI (H2), FC and BI (H4), and QL and BI (H11). The R-Square for BI and UB are 0.845 and 0.467, respectively.

**Table 4:** Structural model

	Path	Β	t- statistics	p-value	Comments
H1	PE—> BI	0.048	0.525	0.600	Rejected (> *p* = 0.05)
H2	EE—> BI	-0.171	1.792	0.074	Rejected (> *p* = 0.05)
H3	SI—> BI	0.186	2.740	0.006	Accepted (< *p* = 0.05)
H4	FC—> BI	-0.055	1.799	0.073	Rejected (> *p* = 0.05)
H5	HM—> BI	0.119	3.617	0.000	Accepted (< *p* = 0.05)
H6	PV—> BI	0.202	3.007	0.003	Accepted (< *p* = 0.05)
H7	HA—> BI	0.614	10.243	0.000	Accepted (< *p* = 0.05)
H8	HA—> UB	0.290	2.447	0.015	Accepted (< *p* = 0.05)
H9	SQ—> BI	0.226	6.477	0.000	Accepted (< *p* = 0.05)
H10	SQ—> UB	0.941	11.817	0.000	Accepted (< *p* = 0.05)
H11	QL—> BI	0.028	0.948	0.343	Rejected (> *p* = 0.05)
H12	QL—> UB	0.357	5.107	0.000	Accepted (< p = 0.05)
H13	BI—> UB	0.331	2.598	0.010	Accepted (< *p* = 0.05)

Performance expectancy = PE, Effort expectancy = EE, Social influence = SI, Facilitating condition = FC, Hedonic motivation = HM, Price value = PV, Habit = HA, Service quality = SQ, Quality of life = QL, Behavioural intention = BI, Use behavior = UB

### Predictive relevance (Q^2^) and model’s effect sizes (f^2^)

Predictive Relevance (Q2) checks if the data points of indicators in the reflective measurement model of endogenous construct can be predicted accurately [147]. In the model, two endogenous constructs, such as behavioural intention and use behaviour, are selected for blindfolding-based cross-validated redundancy measure. Chin [148] suggests that a model demonstrates good predictive relevance when its *Q*^2^ value is larger than zero. It is observed that the proposed model has good predictive relevance for endogenous variables: behavioral intention (*Q*^2^ = 0.757) and use behavior (*Q*^2^ = 0.355).

f^2^ effect size indicates the practical relevance of significant effects of any construct. Cohan’s (1988) [149] suggests that f^2^ values of 0.35, 0.15 and 0.02 are interpreted as large, medium and small effect sizes, respectively. The results show the f2 values, indicating that the exogenous variables in our model have medium to large f2 effect sizes on the endogenous variables (See Appendix 4).

### FsQCA results

#### Necessary conditions for m-health intentions and usage behaviour

Necessary conditions analysis was employed to detect which set of antecedents is necessary to induce m-health intentions and usage behaviour. As discussed in method section (i.e., analytical procedure), a condition with a consistency value greater than 0.9 is subject to necessary conditions [139]. Table 5 illustrates the necessary conditions for m-health intentions. The findings of the necessary analysis revealed that price value (0.922) and habit (0.926) are considered necessary conditions for m-health intentions. In contrast, performance expectancy (0.815), effort expectancy (0.837), social influence (0.834), facilitating condition (0.866), hedonic motivation (0.858), service quality (0.325) and quality of life (0.372) are not necessary conditions for m-health intentions.

**Table 5:** Necessary conditions for intentions to use m-health

Antecedent conditions	Consistency	Coverage
Performance expectancy	0.815	0.956
Effort expectancy	0.837	0.950
Social influence	0.834	0.943
Facilitating condition	0.866	0.733
Hedonic motivation	0.858	0.910
Price value	0.922	0.955
Habit	0.926	0.970
Service quality	0.325	0.964
Quality of life	0.372	0.925

#### Sufficient analysis for m-health intentions

Table 6 illustrates the necessary conditions for usage behaviour of m-health. The findings of the necessary analysis revealed that m-health intentions (0.929) and habit (0.920) are considered necessary conditions for usage behaviour of m-health. In contrast, service quality (0.725) and quality of life (0.663) are not necessary conditions for usage behaviour of m-health.

**Table 6:** Necessary conditions for usage behaviour of m-health

Antecedent conditions	Consistency	Coverage
Behavioural intention to use m-health	0.929	0.264
Service quality	0.725	0.606
Quality of life	0.663	0.461
Habit	0.920	0.272

#### Sufficient analysis for m-health intentions

This section reports the results of sufficient conditions for high and low m-health intentions. FsQCA was performed to investigate the configurational effects of performance expectancy, effort expectancy, social influence, facilitating condition, hedonic motivation, price value, habit, service quality and quality of life on m-health intentions.

As shown in Table 7 (see left side of the Table), fsQCA findings suggested three causal recipes that represent the sufficient conditions leading to high m-health intentions (solution coverage = 0.767 and solution consistency = 0.856). Of the three solutions leading to high m-health intentions, solution 1 is the most desirable solution as it has highest raw coverage of 0.721. The first model suggests that elders, who have high levels of perceived social influence, hedonic motivation, price value, habit and perceived service quality towards m-health, and who have low levels of effort expectancy, facilitating condition towards m-health and quality of life, report their willingness to use m-health.

**Table 7:** Configural effects for predicting high and low behavioural intentions towards m-health

Configural models for predicting high behavioural intentions towards m-health				Configural models for predicting low behavioural intentions towards m-health			
*BI* = *f (PE, EE, SI, FC, HM, PV, HB, SQ and QL)*				~ *BI* = *f (PE, EE, SI, FC, HM, PV, HB, SQ and QL)*			
Configural Models (Sufficient causal recipes)	Raw coverage	Unique Coverage	Consistency	Configural Models (Sufficient causal recipes)	Raw coverage	Unique Coverage	Consistency
**Model 1:** ~ EE*SI* ~ FCI*HM*PV*HA*SQ* ~ QL	0.721	0.010	0.986	**Model 1:** ~ PE* ~ EE*SI* ~ PV* ~ HA *SQ*QL	0.811	0.145	0.949
**Model 2:** PE* ~ EE*SI* ~ FCI*HM*PV*HA*SQ	0.714	0.018	0.986	**Model 2:** ~ PE* ~ EE*SI* ~ PV* ~ HA	0.711	0.031	0.803
**Model 3:** PE*EE*SI*FC*HM*PV*HA* QL	0.546	0.015	0.995				
Solution coverage: 0.767				Solution coverage: 0.900			
Solution consistency: 0.856				Solution consistency: 0.824			

Performance expectancy = PE, Effort expectancy = EE, Social influence = SI, Facilitating condition = FC, Hedonic motivation = HM, Price value = PV, Habit = HA, Service quality = SQ, Quality of life = QL, Behavioural intention = BI, Use behavior = UB

Table 7 (see right side of Table) also presents fsQCA results for two sufficient causal models of unwillingness to use m-health (solution coverage: 0.900 and solution consistency: 0.824). Of the two solutions leading to low m-health intentions, solution 1 is the most desirable solution as it has highest raw coverage of 0.811. The first model recommends that elders, who have high levels of social influence, service quality towards m-health and quality of life, and who have low levels of performance expectancy, effort expectancy, price value and habit towards m-health, report their unwillingness to use m-health.

#### Sufficient analysis for usage behaviour of m-health

This section reports the results of sufficient conditions for high and low usage behaviour of m-health. FsQCA was employed to investigate the configurational effects of m-health intention, service quality, habit and quality of life on usage behaviour of m-health.

As shown in Table 8 (see left side of the Table), fsQCA findings suggested two causal recipes that represent the sufficient conditions leading to high usage behaviour of m-health (solution coverage = 0.971 and solution consistency = 0.854). Of the two solutions leading to high usage behaviour of m-health, solution 1 is the most desirable solution as it has highest raw coverage of 0.913. The first model suggests that elders, who have high levels of m-health intention, habit towards m-health and quality of life, report their high usage behaviour of m-health. Table 8 (see right side of Table) also presents one sufficient causal model of low usage behaviour of m-health (solution coverage: 0.711 and solution consistency: 0.907). The model recommends that elders, who have low levels of m-health intentions, habit and service quality towards m-health, report their low usage behaviour of m-health.

**Table 8:** Configural effects for predicting high and low usage behaviour of m-health

Configural Models for predicting high usage behaviour of m-health				Configural Models for predicting low usage behaviour of m-health			
UB = *f (*BI, SQ, QL and HA)				~ UB = *f (*BI, SQ, QL and HA)			
Configural Models (Sufficient causal recipes)	Raw coverage	Unique Coverage	Consistency	Configural Models (Sufficient causal recipes)	Raw coverage	Unique Coverage	Consistency
**Model 1:** BIN*HA*QL	0.913	0.287	0.879	**Model 1:** ~ BIN* ~ SQ* ~ HA	0.450	0.010	0.972
**Model 2:** BIN*HA*SQ	0.616	0.042	0.766				
Solution coverage: 0.971				Solution coverage: 0.711			
Solution consistency: 0.854				Solution consistency: 0.907			

BI: Behavioural intention towards m-health, SQ: Service quality, QL: Quality of life, HA: Habit and UB: Use behaviour of m-health

## Discussion

The study applied the UTAUT2 model to determine the elderly’s behavioral intention to adopt and use mHealth services in Bangladesh. The study extended the basic UTAUT2 model with two additional variables, quality of life and service quality. The hypotheses proposed in this study have empirical support, and the findings are consistent with the earlier studies on the application of UTAUT in explaining mHealth adoption and usage [17, 150].

The findings of this study indicate that PE and EE were not significant in explaining the BI of the elderly’s mHealth use in the Bangladesh context. The findings apparently contradict previous mHealth literature [17, 87–89, 151, 152]. PE and EE were also found significant factors influencing the adoption of healthcare information technology [90–93, 153, 154]. However, the finding of PE in this study aligns with the finding by Boontarig et al. [91] in the context of Thailand. Their study indicated that the Thai elderlies are aware of technology but not interested enough in the use of mHealth in smartphones. Another possible reason for the rejection of the hypotheses about PE could be the fact that the available mHealth apps have not reached a certain level of maturity that can create enough expectancy about the apps. Besides, it may also happen that the elderlies are now aware of the benefits of using mHealth.

On the other hand, the rejection of the hypothesis about EE can be explained by the findings by Ndayizigamiye and Maharaj [154] and Shareef et al. [155]. The possible explanation of this finding might be the fact that, rather than being used by the elderly themselves, the family members assist the elderly in using mHealth in Bangladesh, where the joint family system is still prevalent. Alternatively, as efficacy expectation is increased through performance accomplishments or learning by doing, the elderlies may not have enough experience of learning by using mHelath services themselves. Furthermore, the study by Vanneste et al. [156] and Liu et al. [157] reported that EE is not significant determinant of BI.

In this study, FC was also not found significant in explaining the elderly’s intention to use mHealth services. This finding is consistent with previous findings by Hoque and Sorwar [41] as that study also aimed to identify the factors that might affect the adoption of mHealth by elderlies in Bangladesh. Additionally, the recent study by Ndayizigamiye et al. [158] supports the finding about FC in this study.

On the contrary, the hypothesis about SI was supported, which is evident in the Bangladesh context as the local socio-economic and family culture influence most decisions taken in a family, especially concerning health-related decisions [88]. More specifically, the elderly’s health related decisions are influenced mostly by family members and social surroundings because of the source of financial support, the elderly's living status, and participants involved in the elderly's health management. A recent study by Shareef et al. [155] also indicates that SI significantly impacts young individuals’ perception of the adoption and usage of mHealth services through SMS in Bangladesh. Also, the study finding by Byomire and Maiga [159] supports the finding that SI has a significant influence on the BI to use mHealth.

The findings of this study confirm that the additional factors HM, PV, and HA of the UTAUT2 model are significant in determining the elderly’s intention to adopt and use mHealth services. Related studies in technology adoption and usage in information system (IS) literature have offered ample support in line with the findings of this study about the impact of HM, PV and HA on adoption and usage of mHealth services [84, 99, 102, 107, 128, 130, 160]. Although Dwivedi et al. [161] previously found PV as significant in Bangladesh considering the cost of mHealth services, now the scenario has somewhat changed in recent times as the costs of mobile phone and the call charge for mHealth services have both reduced substantially over time in Bangladesh. mHealth services have become affordable for mass people for various medical advice from professional doctors with a call charge of only BDT 5 (USD 0.06) per minute (i.e., Grameenphone Tonic).

SQ has been considered an essential element of mHealth [84] and vital in healthcare systems [82]. The study found a significant positive influence of SQ in elderly users’ BI and actual usage of mHealth. This finding aligns with the existing literature where SQ had significant impact on mHealth adoption, usage, and continuance of usage [82, 87, 162]. This study also confirms that QL has positive influence on the UB of mHealth services. This finding is consistent with the findings of a study by [82] on mHealth, where QL had significant impact on users’ continuance intentions for mHealth services. The previous study by Bong, Bergland, and Chen (2019) mentioned that health condition, financial condition, and social relationships and participation are among the eight major dimensions of QL for elderly people. They also mentioned that QL could be affected by a user’s personal beliefs and relationship to the environment. Therefore, the authors safely assume that once an elderly person starts to use mHealth services, the use behaviour of the user can be determined by the QL which in turn depends on various dimensions stated above. Any future study may explain better why QL could significantly influence the use behaviour of the elderlies if the influence of the dimensions of QL on mHealth use bahaviour is empirically tested.

In contrast, surprisingly, QL was found insignificant in explaining the elderly’s BI to adopt mHealth services. This finding is somewhat exceptional to this study, and the reasoning behind such an outcome can be explained from three angles. First, the study primarily investigated how satisfied, healthy, and safe the respondents feel about his/her health-related issues. It is, therefore, logical to have an insignificant relationship between the high-level of QL and the intention to adopt mHealth services. If a respondent is already satisfied with their health issues and health environment, s/he may not be interested in adopting mHealth as an additional tool for managing their health. Second, the price value (PV) of mHealth service is considerably high in Bangladesh as the tariff for calls in mHealth service centers is now affordable. Therefore, it can be assumed that a higher QL is not essential before an elderly can opt for having mHealth services. On the flipside and third, with the improvement of mobile phone networks and the availability of mobile phones at a cheaper price, the usage of mobile phones among the elderly is prevalent in Bangladesh. Such access to a mobile device is argued as a precursor for the elderly's mHealth services usage. Therefore, the QL does not practically belong to the eligibility criteria for BI to use mHealth services by elderlies in the Bangladesh context.

The study also found that BI of adopting mHealth services had a significant positive influence on the elderly’s UB of mHealth services. Previous studies have offered adequate support for such an outcome. For example, Ifinedo [162] found a significant correlation between BI and UB, whereas empirical studies confirmed that higher BI results in a greater UB of a technology [87].

When conducting the PLS Path model analysis, the R2 value, related to research question 2, shows that the theoretical framework (i.e., the extension of UTUAT by QL and SQ) explains 84.5% of the variance in behavioural intention. Further, the findings of fsQCA suggest that elders’ intention to use m-health is high when they have high levels of perceived social influence, hedonic motivation, price value, habit and perceived service quality towards m-health. The results further recommend that elders’ usage behaviour of m-health is high when they have high level of m-health intention, habit towards m-health and quality of life.

Overall, each finding in this study is highly context specific and vital. For example, the findings regarding PE implies that the elderly users are not either well aware of mHealth services in Bangladesh or mHealth services in Bangladesh are not deemed useful enough to create use intention. On the other hand, the insignificant influence of EE and FC implies that there is a need for creating perceptions of EE and FC among elderlies in Bangladesh by letting them use mHealth services themselves without any help from friends and family members. Additionally, the rejection of QL’s influence on BI but the acceptance of QL’s influence on UB importantly inform the researcher that there are necessity future studies for understanding which dimensions of QL (e.g., health condition, financial condition, and social relationships and participation personal beliefs and relationship to the environment) specifically determine the acceptance of mHealth service as well as the use behaviour.

### Theoretical implications

In addition to the basic UTAUT2 model, this study provided valuable insights into two essential factors of mHealth adoption: quality of life and service quality. Therefore, the study adds additional knowledge to the IS research by offering a theoretical framework for adopting and using mHealth in the elderly community in threefold ways. The research contributes to IS literature by first applying the UTAUT2 model to explain mHealth acceptance and usage of elderlies; secondly, it addresses the impact of the quality of life into the default UTAUT2 model and, thirdly, explaining the logical significance of service quality in mHealth acceptance and usage. This study contributes to the healthcare literature by pointing out the most influential factors for elderlies to use mobile health services and enables policymakers and service providers to offer more efficient mHealth services while keeping the significant factors under proper consideration. Finally, our fsQCA analysis provides further insights into m-health intention and usage behaviour. That is, in addition to the overall findings obtained from SEM, fsQCA findings suggest how the intensity of the influencers may contribute to high vs low m-health intention and usage behaviour.

### Managerial implications

The empirical findings provide practical guidelines and suggest essential factors that bear implications in planning, designing, and developing mHealth services for elderlies in Bangladesh. The study findings specific to mHealth service quality will help designing better mHealth tools for universal health coverage by providing insights about how elderlies perceive mHealth service quality and what service quality dimensions are significant to design a successful mHealth intervention. Similarly, the study results show that elderlies' quality of life is an important determinant of mHealth use behavior, which informs health policymakers that well-being and healthy living can act as the preconditions for mHealth’s effectiveness and continued adherence. mHealth service providers should focus on advertisements using reference groups since social influence is an essential predictor of behavioral intention to use mHealth services. mHealth technology should be reliable and easy to use, so the elderlies perceive better control and confidence in using the system. Additionally, the user should be provided with the support of the technology, through training and well as materials and human resources as needed as the facilitating condition has a significant effect on the elderly’s intention to use mHealth service in Bangladesh. Due to the generalized nature of the study, the findings and the theoretical framework can be easily modified to enable planning and implementing mHealth services in other developing nations.

## Conclusions

Lack of understanding of the factors influencing technology adoption might cause digital exclusion of elderly people, specifically in developing countries [163]. To understand the significant factors influencing mHealth adoption and usage in the elderly population of Bangladesh, the study has developed and validated a theoretical framework. The findings suggest that the Social Influence (SI), Hedonic Motivation (HM), Price Value (PV), Habit (HA), and Service Quality (SQ) have significant influence on elderlies’ BI to the adoption and usage of mHealth service. However, the quality of life (QL) was not found significant in explaining the adoption of mHealth services, which is inconsistent with existing literature. Nevertheless, for Bangladesh, the finding is well aligned with the low-cost specifications of mHealth that do not require individuals to possess a high quality of life for availing mHealth services. In the socio-economic context in Bangladesh, quality of life matters for the continued usage of mHealth services but not for the uptake of such service. This is due to quite reasonably priced mHealth services and mobile phones and cellphone networks widely available across the country.

The study extends the conceptual grounding of the UTAUT2 model by adding the impacts of QL and SQ to articulate the adoption and usage behavior of mHealth services in the context of an elderly population. The study findings suggest that the cognitive and contextual traits of the elderly population have implications in explaining the acceptance and usage of mHealth services in a developing country phenomenon. The overall findings may contribute to shaping appropriate policies for designing and implementing mHealth services effectively for elderlies in developing countries.

### Limitations and future research directions

Due to the age-specific characteristic of the sample and a cross-sectional perspective of this study, several limitations prevail in the findings of this study. First, due to the sampling technique used and age-specific characteristics of the targeted samples, there is concern about the findings' generalizability to all populations with various age ranges. However, considering the findings of diverse demographic and socio-economic statuses of the sample drawn from population nationally, the authors argue that the selected sample could be a true representative of the entire elderly population of Bangladesh. To offer a more generic view of the theoretical framework, a further extension of this study could include other age groups of Bangladeshi citizens. Second, the cross-sectional nature of the study could not offer a comparative view of the contingent and causality impact of the users’ experience level before and after the adoption and usage of mHealth services. To uncover a causal relationship among the specified factors, longitudinal data could be used in future studies. In a future study, the use of protection motivation constructs (e.g., Perceived Vulnerability, Perceived Severity, Self-Efficacy, and Response Efficacy) can be considered. Additionally, a future study could more context specific constructs (e.g., social factors and physical infrastructure such as tradition, conformity, power, culture, and government policy).

Also, the study's sample was recruited from public and private hospitals in Dhaka (the capital city of Bangladesh), where patients attend from different geographical areas of Bangladesh. The researchers also attempted to ensure a sample representing the overall elderly population of Bangladesh by examining the respondents' postal codes. Nevertheless, there could be a possibility of systematic and/or random bias in the sample as data were gathered from hospitals in Dhaka city only. Besides, this study sample had low participation from female elderlies, which may hamper the generalizability of results. Therefore, future studies should recruit samples from outside of Dhaka city and more female elderlies to provide richer results. Moreover, differences in cultural dimensions may cause variability in the effectiveness of a health intervention. Therefore, cross-country variation in the sample and cultural attributes in the research model may help future studies offer more comprehensive study findings. Lastly, a further study could investigate the moderating and mediating effects of QL and SQ on the relationships between the dependent and independent variables.

## Data Availability

The datasets generated and analyzed during the current study are stored in our database and will be publicly available.

## Appendix

### Appendix 1: Type factors and hypotheses

**Table:**

Factors	Supporting references	Hypothesis	Measurement Instruments (Adapted for the study’s context)
Performance Expectancy (PE)	[41, 77, 81, 88, 120]	H1: PE has positive impact on the elderly's intention to use mHealth	[88, 89]
Effort Expectancy (EE)	[41, 81, 120, 164]	H2: EE has positive impact on the elderly's intention to use mHealth	[77, 88, 92]
Social Influence (SI)	[41, 81, 93, 120, 164]	H3: SI has positive impact on the elderly's intention to use mHealth	[77, 96, 97]
Facilitating Condition (FC)	[81, 88, 94, 99, 165]	H4: FC has positive impact on the elderly's intention to use mHealth	[100, 101]
Hedonic Motivation (HM)	[81, 101–103, 105, 165]	H5: HM has positive impact on the elderly's intention to use mHealth	[106, 165]
Price Value (PV)	[81, 107]	H6: PV has positive impact on the elderly's intention to use mHealth	[108, 109]
Habit (HA)	[60, 81, 165]	H7: HA has positive impact on the elderly's intention to use mHealth	[110, 111]
		H8: HA has positive impact on the elderly's use behavior of mHealth	
Service Quality (SQ)	[60, 82, 84, 85, 111, 112]	H9: SQ has positive impact on the elderly’s intention to use mHealth	[113–115]
		H10: SQ has positive impact on the elderly’s use behavior of mHealth	
Quality of Life (QL)	[46, 49, 74, 82, 116, 117, 134, 161, 166, 167]	H11: QL has positive impact on the elderly's intention to use mHealth	[73]
		H12: QL has positive impact on the elderly’s use behavior of mHealth	
Behavioral Intention (BI)	[77, 81, 119–121, 164]	H13: BI has positive impact on the elderly's use behavior of mHealth	[81, 119]
Use Behavior (UB)	[81, 120]		[164]

### Appendix 2: Items’ loadings

**Table:**

	BI	EE	FC	HA	HM	PE	PV	QL	SI	SQ	UB
BI1	0.936										
BI2	0.963										
BI3	0.957										
EE1		0.931									
EE2		0.930									
EE3		0.885									
EE4		0.938									
FC1			0.818								
FC2			0.728								
FC3			0.891								
HA1				0.915							
HA2				0.928							
HA3				0.957							
HM1					0.908						
HM2					0.923						
HM3					0.949						
PE1						0.879					
PE2						0.930					
PE3						0.950					
PE4						0.950					
PE5						0.924					
PE6						0.907					
PV1							0.936				
PV2							0.928				
PV3							0.943				
QL1								0.829			
QL2								0.887			
QL3								0.844			
QL4								0.840			
QL5								0.813			
QL6								0.698			
QL7								0.728			
QL8								0.661			
SI1									0.938		
SI2									0.908		
SI3									0.896		
SQ1										0.877	
SQ2										0.956	
SQ3										0.928	
SQ4										0.945	
SQ5										0.938	
SQ6										0.927	
UB1											0.814
UB2											0.933
UB3											0.909
UB4											0.902

### Appendix 3: Variance inflation factors (VIFs)

**Table:**

	VIF
BI1	3.890
BI2	6.379
BI3	5.756
EE1	5.747
EE2	5.414
EE3	4.383
EE4	5.523
FC1	1.564
FC2	1.851
FC3	1.599
H1	3.274
H2	3.605
H3	5.119
HM1	2.882
HM2	3.430
HM3	3.957
PE1	7.648
PE2	9.912
PE3	7.669
PE4	7.535
PE5	5.594
PE6	5.366
PV1	3.702
PV2	3.486
PV3	4.019
QL1	3.090
QL2	3.484
QL3	3.071
QL4	2.904
QL5	2.424
QL6	1.886
QL7	2.068
QL8	1.806
SI1	3.671
SI2	2.547
SI3	2.909
SQ1	3.543
SQ2	9.052
SQ3	5.207
SQ4	7.805
SQ5	6.699
SQ6	5.551
UB1	1.851
UB2	3.939
UB3	4.433
UB4	4.013

### Appendix 4: f2 values

**Table:**

	BI	UB
**BI**		0.036
EE	**0.016**	
FC	**0.014**	
**HA**	0.605	0.036
HM	**0.038**	
PE	**0.002**	
PV	**0.047**	
**QL**	0.003	0.116
SI	**0.038**	
**SQ**	0.124	0.622
UB		
